# Development of a fast curing tissue adhesive for meniscus tear repair

**DOI:** 10.1007/s10856-016-5790-6

**Published:** 2016-11-19

**Authors:** Agnieszka Izabela Bochyńska, Gerjon Hannink, Dennis Janssen, Pieter Buma, Dirk W. Grijpma

**Affiliations:** 1 MIRA Institute for Biomedical Engineering and Technical Medicine and Department of Science and Technology, Department of Biomaterials Science and Technology, University of Twente, Enschede, The Netherlands; 2 Orthopaedic Research Laboratory, Department of Orthopaedics, Radboud Center for Molecular Life Sciences, Radboud University Medical Center, Nijmegen, The Netherlands; 3 Department of Biomedical Engineering, University of Groningen, University Medical Centre Groningen, W.J. Kolff Institute, Groningen, the Netherlands

## Abstract

Isocyanate-terminated adhesive amphiphilic block copolymers are attractive materials to treat meniscus tears due to their tuneable mechanical properties and good adhesive characteristics. However, a drawback of this class of materials is their relatively long curing time. In this study, we evaluate the use of an amine cross-linker and addition of catalysts as two strategies to accelerate the curing rates of a recently developed biodegradable reactive isocyanate-terminated hyper-branched adhesive block copolymer prepared from polyethylene glycol (PEG), trimethylene carbonate, citric acid and hexamethylene diisocyanate. The curing kinetics of the hyper-branched adhesive alone and in combination with different concentrations of spermidine solutions, and after addition of 2,2-dimorpholinodiethylether (DMDEE) or 1,4-diazabicyclo [2.2.2] octane (DABCO) were determined using FTIR. Additionally, lap-shear adhesion tests using all compositions at various time points were performed. The two most promising compositions of the fast curing adhesives were evaluated in a meniscus bucket handle lesion model and their performance was compared with that of fibrin glue. The results showed that addition of both spermidine and catalysts to the adhesive copolymer can accelerate the curing rate and that firm adhesion can already be achieved after 2 h. The adhesive strength to meniscus tissue of 3.2–3.7 N was considerably higher for the newly developed compositions than for fibrin glue (0.3 N). The proposed combination of an adhesive component and a cross-linking component or catalyst is a promising way to accelerate curing rates of isocyanate-terminated tissue adhesives.

## Introduction

Traumatic meniscal tears are common injuries of the knee. They result in pain, swelling and locking of the knee joint, and may eventually result in the development of osteoarthritis [1]. Bucket handle tears are among the most frequently occurring types of tears resulting from the injury [2, 3]. These are extended, longitudinal tears that are not likely to heal spontaneously due to the displacement of part of the torn tissue.

Tissue adhesives are attractive biomaterials to be used for treating meniscus tears [4]. They are already employed in clinical practice for various applications. Cyanoacrylates are used clinically for gluing skin lacerations, fibrin glue for pulmonary leaks and repair of cardiovascular defects, and TissueGlu^®^ for abdominoplasty [5–7]. However, these materials are not suitable for the repair of meniscal tears either due to their cytoxicity or their insufficient mechanical- and adhesive properties. Therefore, there is a growing interest in the development of new tissue adhesives, which are strong, non-toxic, biodegradable and fast-curing.

Isocyanate-terminated polymers are of particular interest, since they have easily tuneable mechanical properties, they attach to the tissue via formation of strong covalent bonds and degrade to non-toxic products [4]. However, a drawback of this class of materials is the relatively long curing time: it can take up to several hours before the adhesive reaches maximum bonding strength. Few strategies have been employed to speed up the curing rates of isocyanate-terminated polymers. Sternberg et al. engaged the use of polymers such as hyaluronic acid, gelatin, chitosan acetate, and chitosan chloride, which were mixed with the isocyanate-terminated adhesive component prior to its application [8, 9]. Another strategy was chosen by Beckman et al*.* and Smith et al. who added catalysts such as 2,2-dimorpholinodiethylether (DMDEE) and 1,4-diazabicyclo [2.2.2] octane (DABCO) to isocyanate-terminated adhesive compounds [10, 11]. They reported that attachment to the tissue can already be achieved after several minutes.

We have recently developed a series of isocyanate-terminated amphiphilic block copolymers for the repair of meniscal tears [12, 13]. These adhesive materials have satisfactory adhesion strengths to meniscus tissue, adequate mechanical properties of the networks after curing, in addition they are biodegradable and compatible with meniscus cells and tissue when used in physiological amounts. However, it takes between 8 and 24 h for these materials to completely cure. This may cause leakage into surrounding tissues and limit their potential clinical application.

The aim of the current study was to develop a fast curing tissue adhesive (glue) based on a previously developed hyper-branched adhesive block copolymer [13, 14]. We describe two ways of accelerating the curing rate of this isocyanate-terminated copolymeric glue. One strategy was to employ spermidine, a natural liquid polyamine of low molecular weight found in living tissues, that acts as a cross-linker. The other strategy was based on the addition of a catalyst to the isocyanate-terminated copolymer, which will accelerate the curing process in the presence of surrounding body fluids. The resulting adhesive compositions were evaluated in terms of their curing rates, and their adhesion strengths to meniscus tissue.

## Materials and methods

### Adhesive components

The reactive isocyanate-terminated hyper-branched adhesive block copolymer was synthesized as previously reported in detail [13]. The adhesive was prepared from polyethylene glycol (PEG, *M*_n_ = 400 g/mol), trimethylene carbonate (TMC), citric acid (CA) and hexamethylene diisocyanate (HDI). The final product was labeled CA-4PEG-(TMC_2_)_2_-HDI. After each synthesis step, the chemical structures of the intermediate reaction products were confirmed by ^1^H NMR spectroscopy (Bruker 400 MHz NMR spectrometer) using CDCl_3_ as the solvent.

### Two-component adhesive with cross-linkers

The two-component adhesive (glue) was composed of the reactive isocyanate-terminated hyper-branched block copolymer (adhesive component) and a cross-linking component. The cross-linking component was a mixture of spermidine (Sigma Aldrich, the Netherlands) and a linear copolymer HO-TMC_2_-PEG_400_-TMC_2_-OH, which was prepared as described previously [12]. Spermidine was chosen as a natural low molecular weight polyamine with low viscosity, which enabled easy mixing with other components. A HO-TMC_2_-PEG_400_-TMC_2_-OH copolymer was designed to contain the same building blocks (PEG, TMC) as the adhesive component. Moreover, a proper molar ratio of isocyanate to amine groups and a proper viscosity of the cross-linking component was ensured.

**Fig. 1:**
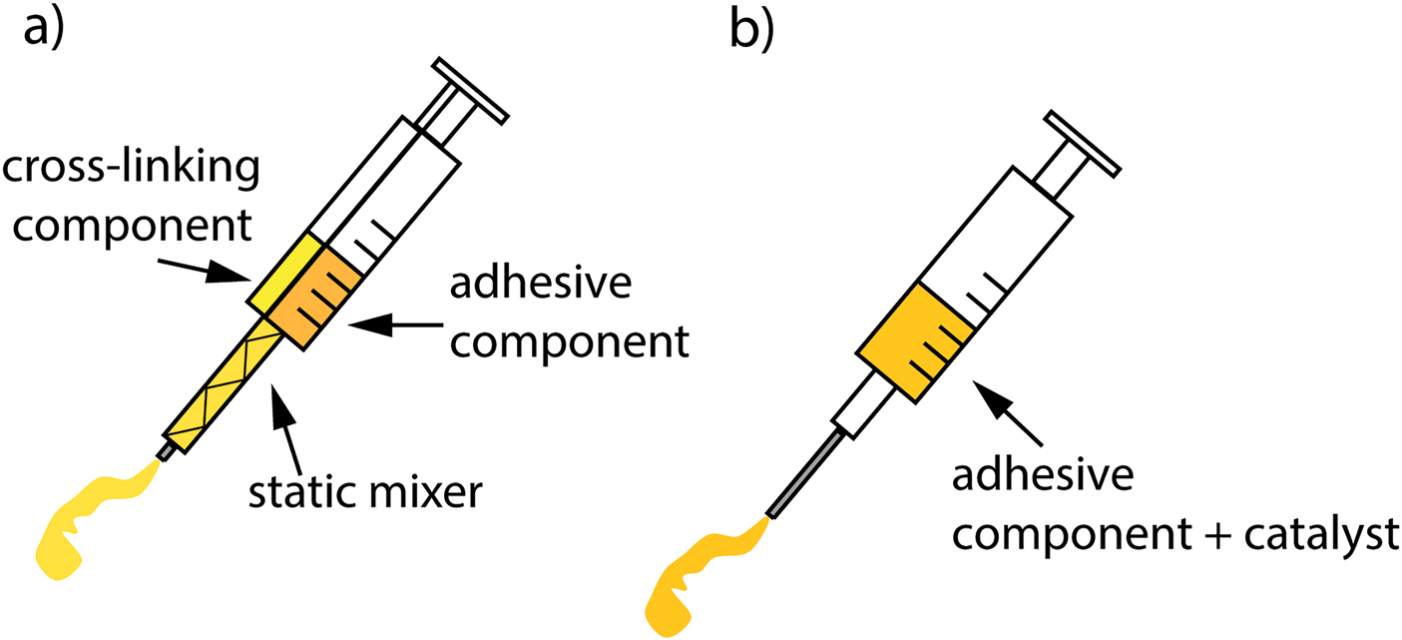
Schematic drawing of the syringe systems used to apply the fast curing adhesives. **a** Double-chamber syringe equipped with a static mixer where the adhesive component and the cross-linking component are mixed during their application, **b** Conventional syringe used to apply a mixture of the adhesive component and a catalyst

In order to obtain an adhesive that cross-links fast and at the same time firmly attaches to the tissue, it was necessary to have an excess of isocyanate to amine groups. Thus, the glue was applied to the tissue with a double-chamber syringe (Sulzer, Switzerland) in which the volume ratio of the adhesive component to the crosslinking component was 4 : 1 (see Fig. 1a). Two different ratios of excess isocyanate to amine groups were evaluated. The amount of spermidine in the cross-linking solution was so adjusted that the ratio of the isocyanate groups of the adhesive component to the amine groups in the cross-linking component was 5 : 1 (spermidine 5 : 1) or 10 : 1 (spermidine 10 : 1) (assuming that the degree of functionalization of the hyper-branched block copolymer with HDI was 100 %). The molar ratio of HO-TMC_2_-PEG_400_-TMC_2_-OH to spermidine in the cross-linking component that was necessary to maintain desired isocyanate groups excess was 2.14 for spermidine 5 : 1, and 4.65 for spermidine 10 : 1.

### Catalysed single-component adhesive

The effect of adding a catalyst to the adhesive component on the curing and the adhesive properties of the cured network was evaluated. The adhesive component (which is a viscous liquid) was placed in a round bottom flask at room temperature (RT) and kept under argon. Subsequently, 2,2-dimorpholinodiethylether (DMDEE, Sigma Aldrich, the Netherlands) or 1,4-diazabicyclo [2.2.2] octane (DABCO, Sigma Aldrich, the Netherlands) was added and the mixture was stirred for 2 h to activate the terminal isocyanate groups of the adhesive component. The amount of added DMDEE was 0.1 wt% of the adhesive component, while the amount of DABCO was equal to 0.034 of the number of isocyanate groups of the adhesive component (assuming 100 % degree of functionalization with HDI). The mixtures of the adhesive component and the catalysts were applied using a conventional 2 mL syringe equipped with a 23G needle (Fig. 1b).

### Curing kinetics of the adhesives

The curing kinetics of the hyper-branched adhesive component and after mixing with a cross-linking component or a catalyst were determined by Fourier transform infrared spectroscopy (FTIR) using a Perkin Elmer Spectrum Two Spectrometer. As the free isocyanate groups react to form a network, the peaks at 2255 cm^−1^ disappear. A drop of the adhesive composition (*n* = 1) was placed on the prism and covered with demineralized water, the spectra were recorded after 0, 5, 15 and 30 min, and after 1, 2, 4, 8 and 24 h. The conversion of isocyanate groups was calculated from the areas of the peaks relative to the peak area at *t* = 0.

### Lap-shear adhesion strength of the adhesive compositions to meniscus tissue

Lap-shear adhesion testing of the adhesives to meniscus tissue was also performed at different curing times. Bovine lateral menisci from a local slaughterhouse were frozen and cut with a cryotome (Microm GmbH, Germany) into strips measuring approximately 0.5 × 10 × 25 mm parallel to the direction of alignment of the circumpherential collagen fibers. The tissue strips were thawed to RT and kept hydrated in water until the gluing experiment. The surfaces of the tissue strips were blotted dry, and the different adhesives were applied to the strips of tissue. The adhesives covered an area of approximately 100 mm^2^. The strips of tissue were then pressed together and immersed in water. The strength of the adhesive bond was measured in a lap shear adhesion test using a Zwick Z020 universal tensile tester in analogy to ASTM F2255-05. The grip to grip separation was 22 mm and the crosshead speed was 50 mm/min. The measurement was performed after 30 min and after 1, 2, 4, 8 and 24 h of applying the glues (*n* = 3). The shear adhesive bond strength (S, expressed in kPa) was determined from the maximum shear force divided by the glued area.

### Evaluation of the adhesive strength of the adhesives in a meniscus bucket-handle tear model

**Fig. 2:**
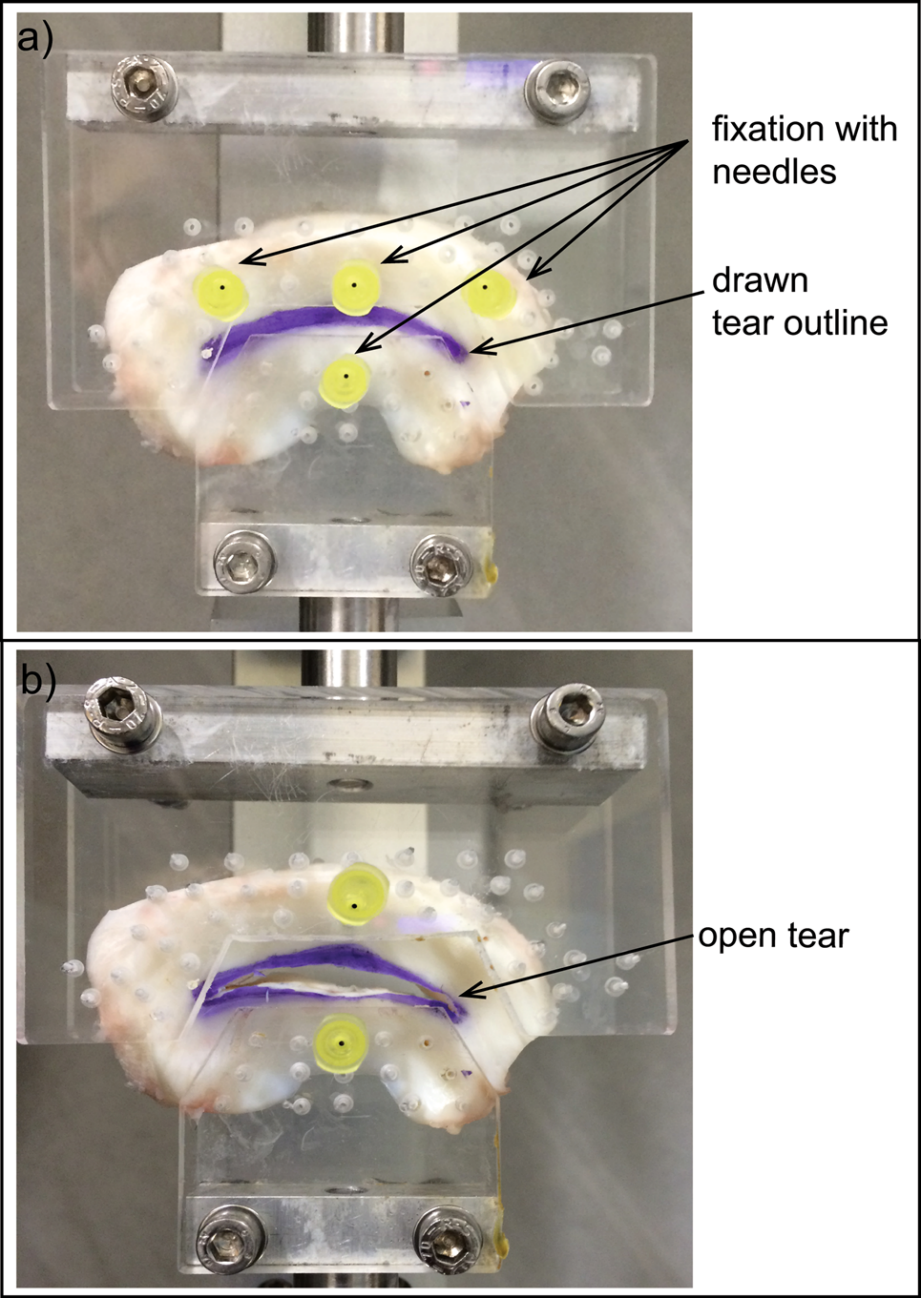
Fixation of a bovine lateral meniscus tissue in a custom-made clamp. **a** Meniscus with a drawn bucket-handle tear outline mounted in the clamp using 4 needles, **b** The two outer needles holding the upper (outer) part of the meniscus were removed, and the bucket-handle tear was opened to allow application of the glues

The adhesive strength of the glues was evaluated in a simulated meniscus bucket-handle tear. Frozen bovine menisci were thawed in water and the outlines of the bucket-handle tears were drawn along the central part of the menisci ending 1 cm from posterior and anterior horns. Subsequently, the menisci were mounted in a custom-made clamp using needles (see Fig. 2a) and placed in a BOSE^®^ Electroforce^®^ setup equipped with a 200 N load cell (BOSE Bose Corp. ElectroForce Systems Group, MN, USA). The outer part of the meniscus was fixed using three needles and the inner part was fixed with a single needle. Then, bucket-handle tears were cut along the drawn outline with a scalpel. The inner part of the meniscus was pulled down at a rate of 5 mm/min to pre-condition the tissue (10 repetitions). The maximum displacement that could be applied with this setup was 6 mm.

The two outer needles fixing the upper (outer) part of the meniscus were removed, the meniscus was now fixated with only one needle holding the upper (outer) part and one needle holding the bottom (inner) part (Fig. 2b). The tissue was then again pre-conditioned by opening and closing the tear (5 repetitions). The meniscal tear was opened again and the tear surface was rasped using a surgical rasp (diamond rasp 45^o^, Acufex, Smith & Nephew). The different adhesive compositions (100 µL) were applied to the surface of the tears using the syringe systems pictured in Fig. 1. In this setup, the performance of the hyper-branched adhesive, the hyper-branched adhesive mixed with spermidine 5 : 1 and the hyper-branched adhesive containing DABCO was evaluated (*n* = 5). As a control, the performance of fibrin glue (a mixture of fibrinogen (100 mg/mL) and thrombin (100 U/mL) in PBS (pH = 7.4), both isolated from bovine plasma, Sigma Aldrich, the Netherlands) was also evaluated (*n* = 3). An experiment in which no adhesive was applied was also performed (*n* = 1). After application of the adhesives, the tear was closed and the meniscus was wrapped with a moist tissue and left to cure at 37 °C.

After approximately 18 h (overnight), bucket-handle tear of the meniscus was opened to evaluate the adhesive strength of the glues. The hyper-branched adhesive component needed longer curing time than other compositions, therefore to have the same protocol during the experiments, the adhesion of all adhesives was tested after overnight curing. Force-displacement curves at 5 mm/min were recorded. After the glue failed, a second force-displacement curve was determined. This curve was used as a baseline for the non-glued meniscus (*n* = 5). The force-displacement curve for a cut meniscus to which no glue was applied (*n* = 1) was also obtained.

The maximum adhesive strength of the glues (the maximum force before failure of the adhesive bond) and corresponding displacement were determined. Additionally, the energy required to open the glued meniscus tears was determined from the area under the force-displacement curve up to the force at failure and the corresponding displacement. The energy to rupture the adhesive bond between the glue and the tissue was calculated as the difference between the energy required to open the glued tear and the area under the baseline curve up to the force at failure and the corresponding displacement (Matlab, The MathWorks, USA).

## Results

Analysis by ^1^H-NMR after each synthesis step and FTIR confirmed that the adhesive hyper-branched block copolymers were successfully synthesized [12, 13].

### Curing kinetics of the adhesive compositions

**Fig. 3:**
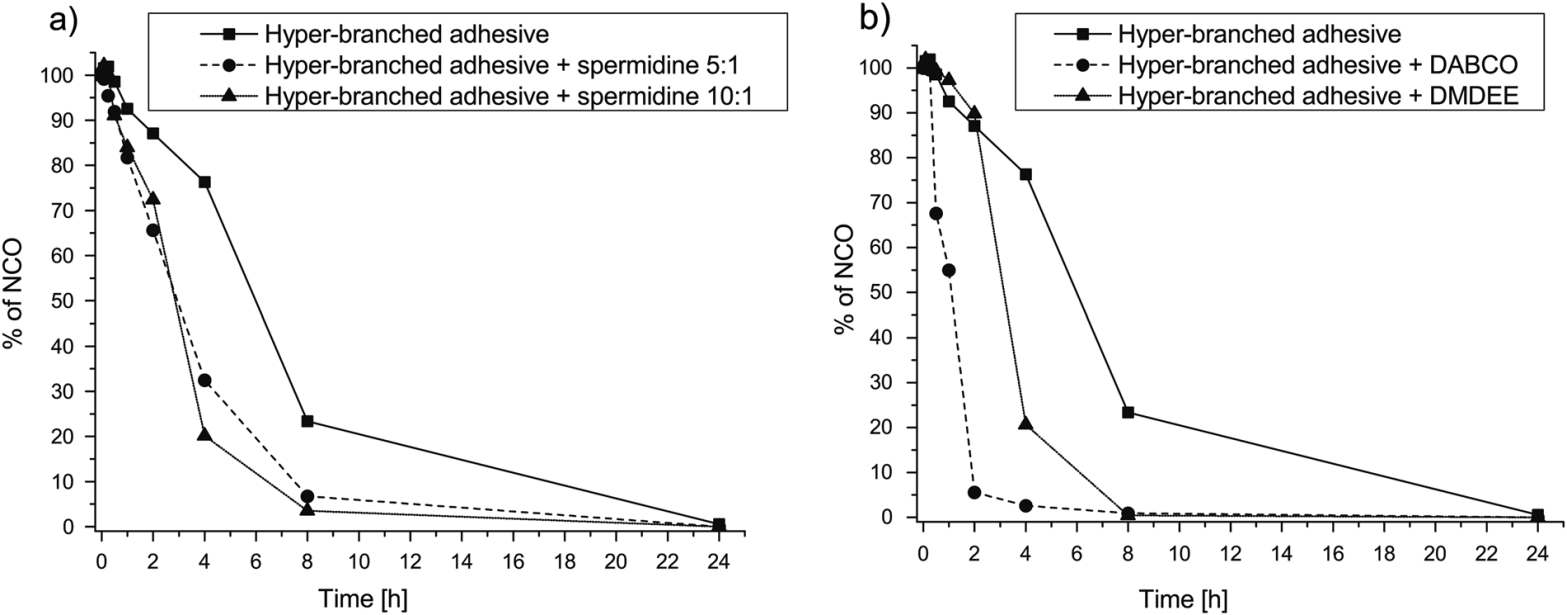
Changes in the amount of unreacted isocyanate groups determined by FTIR during curing by reaction with water of **a** the hyper-branched adhesive component to which cross-linking compositions were added, and **b** the hyper-branched adhesive component to which catalysts were added

The curing kinetics of the hyper-branched adhesive component and those of the hyper-branched adhesive component after mixing with a cross-linking component and a catalyst were determined by FTIR spectroscopy. The disappearance of the peak corresponding to free NCO groups was followed in time, and the conversion of free isocyanate groups upon curing with water was determined (see Fig. 3).

In the hyper-branched adhesive component, 76.3 and 23.3 % unreacted isocyanate groups remained after 4 and 8 h reaction with water, respectively. After 24 h, all isocyanate groups had reacted. By mixing the hyper-branched adhesive with the cross-linking compositions, the rate of curing accelerated significantly: after 4 h the remaining unreacted isocyanate groups were 32.5 and 20.1 % for the compositions with spermidine 1 : 10 and spermidine 1 : 5, respectively. After 8 h these values were 3.5 and 6.7 %, respectively. For all compositions, all isocyanate groups had reacted after 24 h.

When the DMDEE catalyst was added to the hyper-branched adhesive component, its curing rate was similar to that after adding the cross-linking composition. The remaining unreacted isocyanate groups were 20.6 and 0.4 % after 4 and 8 h, respectively. Addition of DABCO to the hyper-branched adhesive component resulted in a much faster curing rate. Already after 2 h the amount of remaining isocyanate groups was only 5.5 %, while after 8 h all isocyanate groups had reacted.

### Lap-shear adhesion strength of the adhesive compositions to meniscus tissue

**Fig. 4:**
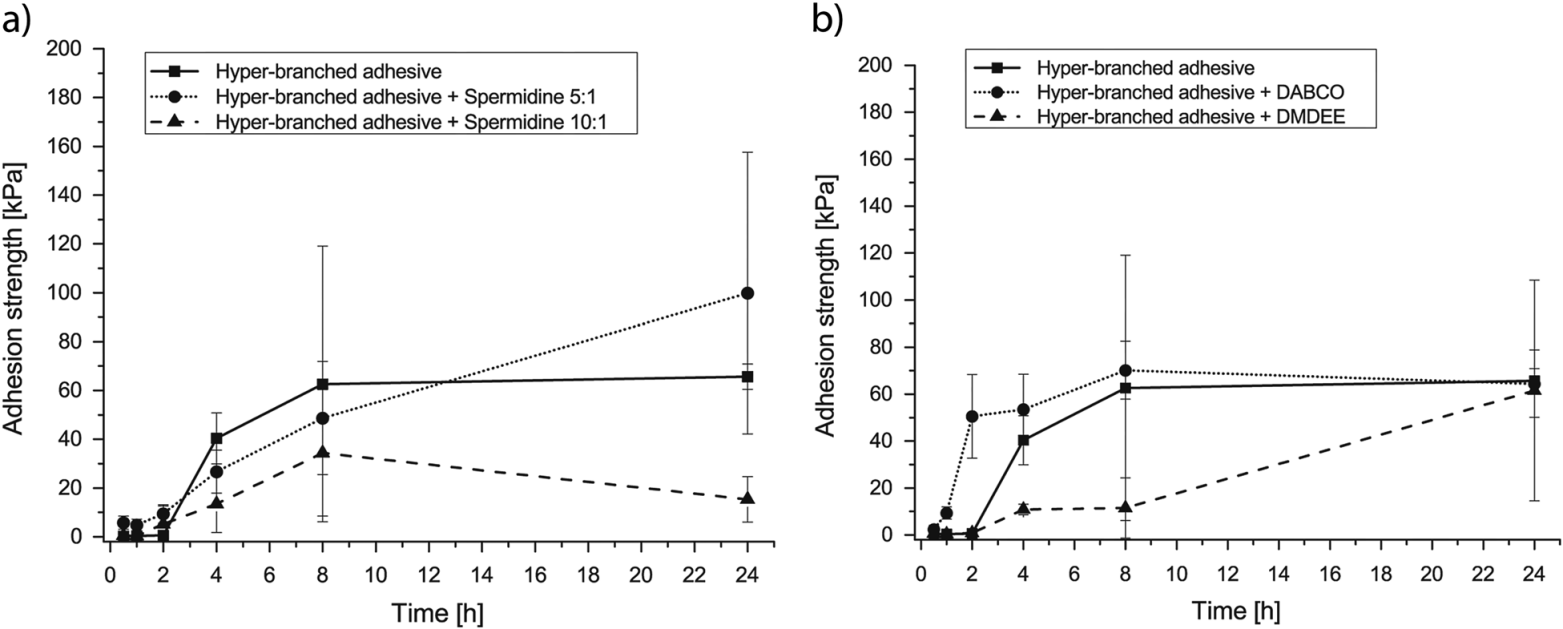
Evolution of the shear-adhesion strength of the different adhesive compositions to meniscus tissue during curing by reaction with water of **a** the hyper-branched adhesive component to which cross-linking components were added and **b** the hyper-branched adhesive component to which catalysts were added. The data are presented as mean ± standard deviations

The shear adhesion strength of the hyper-branched adhesive component and that of the hyper-branched adhesive component after addition of the cross-linking component and the catalysts was determined at different curing times (Fig. 4). The adhesive strength of the hyper-branched adhesive was already close to its maximum value after 8 h (62.6 ± 56.4 kPa) and reached 65.7 ± 5.2 kPa after 24 h. After mixing the hyper-branched adhesive component with spermidine 5 : 1, the determined adhesive strength was 48.7 ± 23.2 and 99.9 ± 57.7 kPa after 8 and 24 h, respectively. The addition of the spermidine 10 : 1 resulted in a lower ultimate adhesive strength of 15.3 ± 9.3 kPa after 24 h.

When the DMDEE catalyst was added to the hyper-branched adhesive, the adhesive strength was relatively low initially; after 4 and 8 h values of 10.9 ± 2.3 and 11.5 ± 12.8 kPa, respectively, were determined. However, after 24 h, the adhesive strength reached 61.5 ± 46.9 kPa. Addition of the DABCO catalyst to the reactive hyper-branched oligomer resulted in already reaching relatively high adhesive strengths after 2 h of application (50.5 ± 14.9 kPa), ultimately reaching 64.4 ± 14.3 kPa after 24 h.

### Adhesion in a bucket-handle meniscal tear model

**Fig. 5:**
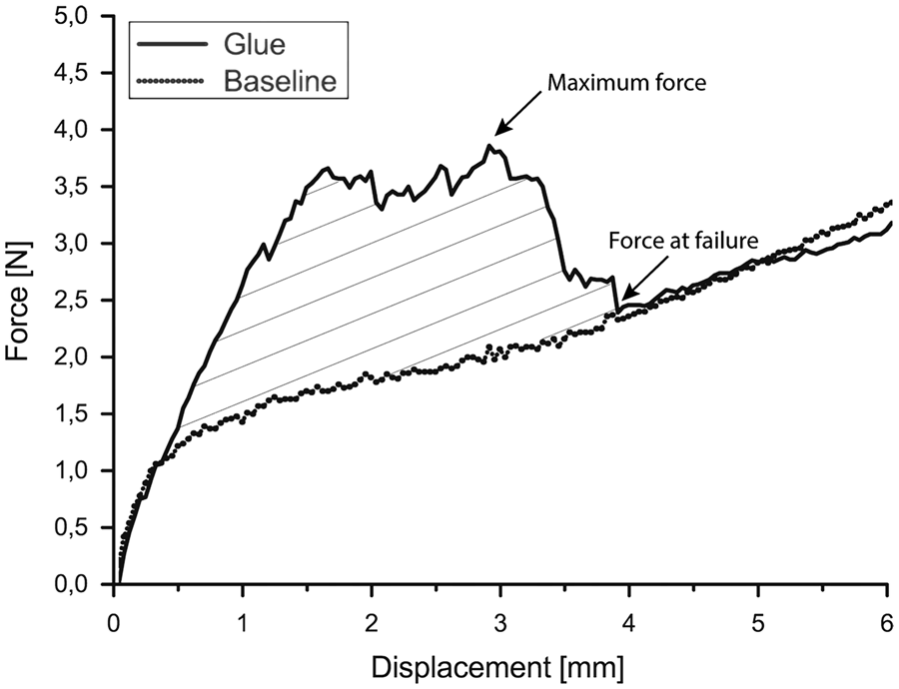
Characteristic force-displacement curves obtained during evaluation of the adhesive strength of the adhesives in a bucket-handle meniscus tear model. The figure shows the behavior of the reactive hyper-branched adhesive component during opening of the glued tear and that of the reference baseline curve obtained after the adhesive failed

The adhesive properties of the hyper-branched adhesive component, and the composition after mixing with spermidine 5 : 1 and DABCO were evaluated in a meniscal bucket-handle tear model. The average length of the tear was 45.0 ± 3.8 mm and the average tear surface area was 353.4 ± 53.3 mm^2^. A characteristic example of the recorded force-displacement curves is shown in Fig. 5.

The maximum adhesive strength of the adhesive component and that of the adhesive to which cross-linking composition and a catalyst were added was between 3.2 ± 0.47 N for the composition with DABCO, and up to 3.7 ± 0.96 N and 3.7 ± 1.5 N, respectively, for the hyper-branched adhesive component alone and the hyper-branched adhesive component to which spermidine 5 : 1 was added (Table 1). The displacement at which the maximum force was reached was between 1.6 ± 0.4 N for the composition with spermidine 5 : 1, and up to 2.8 ± 0.6 mm for the composition with DABCO. It was not possible to determine a maximum adhesive strength of the fibrin glue, as its force-displacement curve was similar to that of a non-glued torn meniscus and it did not show a clear maximum. Thus the highest force recorded during opening the bucket-handle tear glued with fibrin glue was the force of 0.3 ± 0.04 N reached at the maximum displacement of 6 mm. A single meniscus with a simulated tear, but without application of glue was tested as well. It was found that the force-displacement curve was very similar to the baselines determined after failure of the glues in the glued menisci.

**Table 1:** Adhesion testing of different compositions of the reactive isocyanate-terminated hyper-branched adhesives using a bucket-handle meniscus tear model

Glue	Maximum adhesive force [*N*]	Displacement at maximum force [mm]	Energy required to open glued tear [mJ]	Energy required to detach adhesive [mJ]
Hyper-branched adhesive	3.7 ± 0.97	2.4 ± 0.96	11.0 ± 5.2	6.1 ± 3.5
Hyper-branched adhesive + spermidine 5:1	3.7 ± 1.5	1.6 ± 0.4	5.5 ± 3.7	3.2 ± 2.1
Hyper-branched adhesive + DABCO	3.2 ± 0.47	2.8 ± 0.6	10.3 ± 2.1	4.8 ± 1.1
Fibrin glue	0.3 ± 0.04*	6.0*	1.6 ± 0.1**	0

Data are presented as mean ± standard deviation

* The value is the force at the maximum displacement of 6 mm, a maximum force was not reached

** These values are the areas under the curve for displacement from 0 to the maximum displacement of 6 mm

The energy necessary to open a glued meniscus tear was determined from the area under the force-displacement curves of the glued menisci. The highest values were determined for the hyper-branched adhesive component (11.0 ± 5.2 mJ). The energies calculated for compositions with spermidine 5 : 1 and DABCO were slightly lower: 5.5 ± 3.7 mJ and 10.3 ± 2.1 mJ, respectively. For all compositions, the required energy to open the glued tear was pointedly higher than that for the fibrin glue and the non-glued control.

By subtracting the areas under the force-displacement curves of the baseline curves from the force-displacement curves of the glued tears, the energy required to break the adhesive bond between the glue and meniscus could be determined (Fig. 5). The highest values were determined for the hyper-branched adhesive component (6.1 ± 3.5 mJ), followed by the values for the composition with DABCO (4.8 ± 1.1 mJ) and spermidine 5 : 1 (3.2 ± 2.1 mJ). For the fibrin glue this energy was determined to be 0, as its force-displacement curve overlapped the baseline curve.

## Discussion

Two methods to accelerate the curing rates of isocyanate-terminated hyper-branched copolymers were evaluated. In one of the employed strategies a two-component adhesive formulation consisting of a hyper-branched adhesive component and a cross-linking mixture with an amine group-containing compound was used. It is known that amine groups have up to 50 times higher reactivity towards isocyanate-groups than hydroxyl groups, and using such composition will allow the glue to cure within significantly shorter times [8, 9, 15]. The adhesive was applied using a double-chamber syringe equipped with a static mixer. In this way, the two components can be mixed immediately before application on the tissue. For this to occur efficiently, similar viscosities of the components are required. As it is also necessary to maintain the correct (excess) stoichiometric balance between isocyanate groups and amine groups, the spermidine cross-linker was diluted with a low molecular weight hydroxyl group-terminated HO-TMC_2_-PEG_400_-TMC_2_-OH block copolymer. In this manner correct viscosities and ratios of isocyanate groups to amine groups were obtained.

A second strategy to accelerate the curing rate of the adhesive component was the use of catalysts DMDEE or DABCO. These catalysts have been shown to significantly increase the curing rates of isocyanate-terminated adhesives [10, 11].

Both the addition of a cross-linking component and catalysts accelerated the curing rates of the hyper-branched adhesive component (Fig. 3). The curing rate was most increased when DABCO was added to the hyper-branched adhesive, after only 2 h nearly 95 % of the isocyanate groups had already reacted to form a polyurethane network. These results are consistent with literature, where significant increases in curing rates of the isocyanate adhesives after addition of catalysts have been reported [10, 11]. Formulating the reactive hyper-branched adhesive with a cross-linking component resulted in slightly lower curing rates. However, the extruded reacting mixture could be precisely placed on the tissue as a result of its high viscosity. Once placed, it did not flow and would probably not leak into the surrounding tissues when used in a clinical application.

The values of the adhesive strength of the adhesive component and those of the mixture of the adhesive component with spermidine 5 : 1 determined in lap shear tests were similar. However, when the reactive hyper-branched adhesive was mixed with spermidine 10 : 1, the resulting ultimate adhesive strength was lower. It seems that in the latter case the diluent concentration was too high and hampered the adhesion process. When catalysts were added to the adhesive component, the ultimate tensile strengths of the different cured compositions were similar. This indicates that the faster network formation did not hinder the attachment of the adhesives to the tissue, and that the number of remaining unreacted isocyanate groups available was sufficient to allow adhesion of the curing glues to the tissue. Nevertheless, the adhesion strength values of all investigated compositions (except when spermidine 10 : 1 was used) determined in a lap-shear test to meniscus tissue were significantly higher (60–100 kPa) than values reported for the clinically used fibrin glue adhesive (10–20 kPa) [13]. Even though Dermabond^®^ remains the strongest clinically available tissue glue, its application is limited to its use as a topical skin adhesive due to its toxicity.

The adhesive composition to which spermidine 5 : 1 had been added, and the adhesive component mixed with DABCO showed the most promising results in lap-shear adhesion testing and their curing times were considerably shorter than for the adhesive component alone. Thus, these two compositions were further evaluated as adhesive formulations in the bucket-handle meniscus tear model, as bucket handle tears are among the most commonly occurring types of traumatic meniscus injuries. They are of great clinical relevance, since they result in locking of the knee joint [3]. The performance of the two chosen compositions was compared with that of the hyper-branched adhesive component only and fibrin glue. The torn meniscus was preconditioned, glued, kept moist and left overnight to allow the glues cure, after which the adhesive test was performed.

It was observed that during the several hours the meniscus was mounted in the clamp, its mechanical properties had changed and the force needed to open the tear decreased (data not shown). This was probably due to relaxation, which is well-known to occur in biological tissues [16–18]. Also, even though the tissue was wrapped with a moist fabric, it could have been not sufficiently hydrated [19, 20]. Therefore, to be able to assess the effect of gluing the meniscus tears with the different adhesive compositions, comparisons were made with “baseline” force-displacement curves obtained directly after testing the glued menisci to failure. We showed in a single experiment that a meniscus in which a tear was cut (but not glued) and evaluated under the same conditions, displayed similar mechanical properties as those that had been glued and tested to failure. This implies that application of the glues did not affect the mechanical characteristics of the meniscus tissue.

The force necessary to rupture the adhesive bond between the glue and the meniscus and open the bucket-handle tear for the isocyanate-terminated hyper-branched component alone was similar to that after mixing with spermidine 5 : 1 or DABCO. The determined forces were considerably higher than when fibrin glue was employed. Apparently, mixing-in of a cross-linking composition and catalyst to the adhesive do not substantially hamper the adhesive strength of the cured network.

The adhesive strength of the adhesive compositions (3.2–3.7 N) should be sufficient to hold the torn meniscus together during the period of healing, as it has been reported that in a cadaver knee model the distraction forces acting on a torn meniscus do not exceed 5–10 N when a load of 300 N is applied [21, 22]. During the recovery period after surgery, the load on the knee would be even lower. From this it follows that the developed adhesive compositions should be able to perform adequately in the clinic. The simulated lesion was made with a scalpel and even though a mechanical rasp was used to refresh the wound and expose collagen fibers as done in clinical practice [23, 24], a meniscus tear that occurs spontaneously will be much rougher and more fibrillated. In gluing such a tear, better attachment of the adhesive due to larger surface areas and more extensive mechanical interlocking can be expected [25].

The energy required to open the glued meniscus tears was pointedly higher for the developed adhesive formulations than for fibrin glue and the control. The force-displacement curves indicated that these adhesives did not fail immediately when forces close to the maximum values were reached, but were able to maintain an adhesive bond for several more millimeters of applied displacement. The force-displacement curve of the meniscus tear model in which fibrin glue was used was identical to that of the baseline, indicating that fibrin glue had no effect in keeping the edges of meniscus tears together.

## Conclusions

New, rapidly curing adhesive systems composed of a reactive isocyanate-terminated hyper-branched adhesive component and a cross-linking composition or catalyst are promising biomaterials for the repair of meniscal tears. The addition of a cross-linking composition or a catalyst to the hyper-branched adhesive component resulted in considerably accelerated curing rates, without notably compromising the adhesive properties to the tissue. The adhesive strengths evaluated in a meniscus bucket-handle lesion model were found to be much higher than that of fibrin glue. Further studies are needed to confirm the biocompatibility of these systems with cells and tissues in vitro and in vivo.
